# Occupational Stress Management in Nursing Home Helpers: Specialized Practice in Mental Health and Psychiatric Nursing

**DOI:** 10.1192/j.eurpsy.2026.11518

**Published:** 2026-07-31

**Authors:** M. J. Barata, C. Laranjeira

**Affiliations:** 1 School of Health Sciences; 2 Centre for Innovative Care and Health Technology, Polytechnic University of Leiria, Leiria, Portugal

## Abstract

**Introduction:**

Demographic aging has led to a significant demand for institutionalization, placing high demands on Nursing Homes. In this context, direct-action aides play a central role in providing care. However, they are exposed to demanding working conditions characterized by physical and emotional overload, low wages, lack of career advancement, rotating shifts, and lack of recognition. These are significant psychosocial risks that increase occupational stress and have a direct impact on the mental health of professionals.

**Objectives:**

To empower nursing home helpers with coping strategies to reduce occupational stress levels through specialized practice in Mental Health and Psychiatric Nursing.

**Methods:**

A pre-experimental, single-group study was chosen, with pre- and post-intervention assessments taking place over a period of approximately three months. The sample was intentional and selective, comprising eight nursing home helpers. The training program, consisting of seven group sessions, was based on the principles of psychoeducational intervention and the cognitive-behavioral sequence. The data collection instrument consisted of a sociodemographic and professional questionnaire; the 10-item Kessler Psychological Distress Scale (K10); the MHPK-10 Scale; the Occupational Stress Questionnaire – General Version (OQSO-VG); and, finally, the Brief COPE Questionnaire.

**Results:**

After the intervention, there was a significant increase in knowledge of the factors that promote mental health (before=3.21±0.87; after=4.31±0.62; p<0.05). According to the results obtained, there were no statistically significant results in perceived general stress and global stress, but significant reductions were observed in the subscales “Relationship with Managers” and “Relationship with Colleagues” (p<0.05). Regarding coping strategies, significant improvements were observed in the subscales “Planning”, “Use instrumental support”, “Religion”, and “Self-distraction” (p<0.05), reflecting greater use of adaptive strategies.

**Conclusions:**

The results highlight the need to invest in the mental health of this professional group, promoting well-being and adaptive coping strategies to prevent occupational stress. Therefore, it is essential to invest in ongoing training that reinforces positive mental health literacy. From an organizational perspective, the importance of professional development through a set of measures (e.g., emotional salaries) stands out, capable of strengthening motivation, job satisfaction, and the quality of care provided.

**Disclosure of Interest:**

None Declared

